# Association of Seaweed Consumption with Metabolic Syndrome and Its Components: Findings from the Korean Genome and Epidemiology Study

**DOI:** 10.3390/foods11111635

**Published:** 2022-06-01

**Authors:** Haeun Park, Kyung Won Lee, Dayeon Shin

**Affiliations:** 1 Department of Food and Nutrition, Inha University, Incheon 22212, Korea; phepj1017@naver.com; 2 Department of Home Economics Education, Korea National University of Education, Cheongju 28173, Korea

**Keywords:** seaweed, laver, metabolic syndrome, Korean Genome and Epidemiology Study (KoGES)

## Abstract

This study aimed to investigate the association between seaweed consumption and the odds of developing metabolic syndrome in middle-aged and elderly Koreans. The study included 5777 adults aged 40–69 years from 2001 to 2002 in the Ansan and Ansung cohorts of the Korean Genome and Epidemiology Study. Data on the consumption of seaweed, including laver and kelp/sea mustard, were obtained using a semiquantitative food frequency questionnaire. Multivariable logistic regression models were used to assess the association between seaweed consumption and the odds of developing metabolic syndrome and its components. Women in the highest tertile of laver consumption had lower odds of developing metabolic syndrome than those in the lowest tertile (adjusted odds ratio [AOR]: 0.70; 95% confidence interval [CI]: 0.54–0.92). Both men and women in the highest tertile of laver consumption had lower odds of abdominal obesity than those in the lowest tertile (AOR: 0.64, 95% CI: 0.42–0.98 for men; AOR: 0.53, 95% CI: 0.39–0.72 for women). These findings suggest that laver consumption is inversely associated with the odds of developing metabolic syndrome and abdominal obesity in Korean adults.

## 1. Introduction

Metabolic syndrome is characterized by three or more of the following conditions: abdominal obesity, hypertriglyceridemia, low high-density lipoprotein (HDL) cholesterol levels, hypertension, and hyperglycemia [1,2]. As the prevalence of obesity continues to increase, the prevalence of metabolic syndrome also increases [3,4,5]. In Korea, the overall prevalence of metabolic syndrome has increased by approximately 3% over 10 years, from 21.6% in 2007 to 24.5% in 2017 (men, 28.1%; women, 18.7%) [6]. As metabolic syndrome may also increase the risk of type 2 diabetes and cardiovascular diseases, its management and treatment are important [7,8]. Accordingly, investigations are needed to identify modifiable risk factors for metabolic syndrome that can help prevent the disease.

Metabolic syndrome is caused by poor lifestyle management, such as an unhealthy diet, smoking, excessive alcohol consumption, and lack of physical activity [9,10]. Among middle-aged men, those who exercised <5 times a week were approximately 1.5 times more likely to develop hypertriglyceridemia, have low HDL cholesterol levels, and develop abdominal obesity than those who exercised at least five times a week [11]. In addition, current smoking status and alcohol consumption are significantly associated with individual parameters of metabolic syndrome, including obesity, triglyceride levels, insulin levels, and the score for the homeostatic model assessment of insulin resistance [12,13]. It has been reported that a healthy diet is the most important lifestyle factor. Previous studies have reported that those who practice healthy dietary habits have significantly lower prevalence rates of abdominal obesity and metabolic syndrome than those who do not [14]. A healthy diet includes an adequate intake of fat, a low intake of sodium, and sufficient consumption of fruits and vegetables, including seaweed [15]. Seaweed is widely consumed by individuals in East Asian countries—especially in Korea, China, and Japan [16,17]. According to an analysis of the recent Korea National Health and Nutrition Survey, the daily seaweed consumption per person was 3.5 g (3.7 g for men and 3.2 g for women) [18]. Laver (*Porphyra sp.*; red algae), sea mustard (*Undaria pinnatifida*; brown algae), kelp (*Laminaria sp.*; brown algae), *Enteromorpha* (green algae), *Capsosiphon fulvescens* (green algae), and *Hizikia fusiforme* (brown algae) have also been reported to be consumed by these populations [19]. The average daily intake differed depending on the type of seaweed: 1.04 g of laver, 0.94 g of kelp, 0.77 g of sea mustard, 0.33 g of *C. fulvescens*, 0.30 g of *Enteromorpha*, and 0.12 g of *H. fusiforme* [20]. Seaweed contains compounds such as polysaccharides, peptides, and carotenoids, which play a role in controlling obesity and metabolic syndrome [21,22]. Polysaccharides in seaweed have been identified as prebiotic fibers that can be fermented to produce short-chain fatty acids, which help prevent metabolic syndrome by changing the composition of the gut bacteriome and increasing the production of short-chain carboxylic acids [23,24,25]. Extracts of *Porphyra dentata* play a role in preventing obesity by exhibiting dual-generative inhibitory properties in 3T3-L1 cells [26]. In addition, the concentration of *P. dentata* extract was significantly associated with the suppression of obesity-induced gene expression in 3T3-L1 cells [26]. Functional peptides—another component of seaweed—are known to inhibit the activity of angiotensin-converting enzymes, which lower blood glucose levels and blood pressure [27,28]. Previous experiments using rat models have demonstrated the beneficial effects of seaweed extracts, such as fucoidans and polysaccharides, on metabolic syndrome and its components. Seaweed extracts reportedly inhibit lipid accumulation in the blood and exert an anti-obesity effect by reducing the weight and mass of adipose tissues. In addition, seaweed and its polysaccharides exert anti-hyperlipidemic and anti-hypertensive effects, which are potentially useful for preventing or managing cardiovascular diseases [29]. However, a previous animal study reported that levels of HDL cholesterol and triglycerides increased, but with no change in LDL cholesterol levels, when rats were fed a diet containing various types of seaweed, such as *Ulva* spp. and *Hypnea charoides* [30].

However, little is known about the association between seaweed consumption and metabolic syndrome or its components in Korean adults. Therefore, this study aimed to examine the association between seaweed consumption and metabolic syndrome among middle-aged and elderly adults in Korea using the Korean Genome and Epidemiology Study (KoGES).

## 2. Materials and Methods

### 2.1. Study Participants

This study used data from the Ansan and Ansung cohort studies of KoGES which was designed to identify the risk factors for chronic diseases in Koreans and collects epidemiological data on health and lifestyles [31]. The Ansan and Ansung cohort studies began with men and women, aged 40–69 years, living in Ansan (an industrialized community) and Ansung (a rural area) in 2001, respectively. The KoGES recruited survey participants from 2001 to conduct repeated follow-up investigations until 2019, to collect information on metabolic factors, food frequency questionnaires (FFQs), and other variable data.

**Figure 1:**
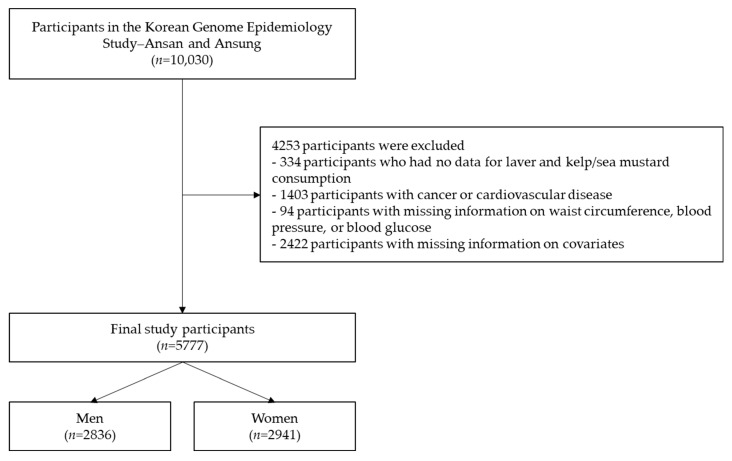
Process flowchart outlining the steps of this analysis.

For this study, of the total 10,030 potential participants, the following were excluded: those without laver or kelp/sea mustard intake data (*n* = 334); those with cancer or cardiovascular disease (*n* = 1403); those without waist circumference, blood pressure, or blood glucose values (*n* = 94); and those without information on covariates, including alcohol consumption, smoking status, education level, income level, body mass index (BMI), and physical activity (*n* = 2422). Of the remaining 5777 participants, 2836 (49.1%) were male (Figure 1). The study protocol was reviewed and approved by the Institutional Review Board (IRB) of Inha University on 31 January 2020 (IRB No. 200129–1A).

### 2.2. Assessment of Metabolic Syndrome and Its Components

The dependent variables in this study were the presence of metabolic syndrome and the presence of its individual components. Participants were confirmed to have metabolic syndrome if they had more than three of the following: abdominal obesity, elevated triglycerides, elevated fasting glucose, elevated blood pressure, and low HDL cholesterol. Waist circumference was measured using a tape placed horizontally around the middle area between the rib and the iliac ridge. The average measurement from a total of three measurements was calculated. Men with a waist circumference ≥ 90 cm and women with a waist circumference ≥ 80 cm were defined as having abdominal obesity [32]. Triglyceride, fasting blood glucose, and HDL cholesterol levels were measured using an ADVIA 1650 Clinical Chemistry Analyzer. Participants were classified as having hypertriglyceridemia if their triglyceride level was ≥ 150 mg/dL [3,33]. Participants were classified as having diabetes if their fasting blood glucose level was ≥ 126 mg/dL, if they were currently being treated for diabetes using hypoglycemic agents or insulin, or if they had a history of receiving treatment for diabetes [34]. Men with an HDL cholesterol level < 40 mg/dL and women with an HDL cholesterol level < 50 mg/dL were classified as having low HDL cholesterol levels [3,33]. Blood pressure was measured at least twice at every visit in a seated position, with the arms positioned at the level of the heart. Elevated blood pressure was defined as systolic blood pressure > 130 mmHg, diastolic blood pressure > 85 mmHg, history of hypertension, blood pressure medication, or current treatment for hypertension.

### 2.3. Assessment of Seaweed Consumption

Seaweed consumption was assessed by measuring the intakes of laver, kelp/sea mustard, and total seaweed. The total seaweed consumption was determined by combining the average daily intake of laver and kelp/sea mustard, which was determined using a semi-quantitative FFQ. The FFQ displays a list of 106 food items frequently consumed by Koreans and examines their frequency and amount of consumption over the past year. The standard amount of laver per intake was one sheet (large), while that of kelp/sea mustard was one bowl of soup.

The intake frequency was categorized into nine timeframes (i.e., none, almost once a month, 2–3 times a month, 1–2 times a week, 3–4 times a week, 5–6 times a week, once a day, twice a day, and 3 times a day). The portion size of each food item was categorized as small (half the standard portion size), medium (one standard portion size), or large (two standard portion sizes). In this study, the frequency of intake was converted to daily intake. The intake amount was converted into grams. The study participants were classified into three groups based on their average daily intake (g) of seaweed, laver, and kelp/sea mustard.

### 2.4. Assessment of Other Variables

BMI was calculated by dividing body weight (kg) by the square of height (m). Education level was divided into three categories: elementary/middle school, high school/professional college, and university. Income level was also categorized into three categories: <1 million won, between 1–3 million won, and >3 million won per month. Smoking status and alcohol consumption were classified as none, past, or current. Physical activity levels were evaluated to determine time spent sedentary and the time spent participating in low-, medium-, or high-intensity activities. The average metabolic equivalent of task (MET) value for each activity was calculated by assigning the MET values to each degree of physical activity. Activity time was converted to hours and multiplied by the average MET value. All values were combined to obtain the total number of MET-hours per day.

### 2.5. Statistical Analyses

General and clinical characteristics and dietary intake of the study participants according to tertiles of seaweed consumption were compared using analysis of variance for continuous variables and chi-squared tests for categorical variables. Multivariable logistic regression analysis was used to calculate the adjusted odds ratios (AORs) for the association between total seaweed, laver, and kelp/sea mustard consumption and metabolic syndrome and its components, after controlling for covariates. The covariates included age; sex; region; alcohol consumption; smoking; education and income level; BMI; physical activity; total energy intake; and protein, fat, carbohydrate, and fiber intake. The *p*-value for trend was calculated by modeling the median values of seaweed, laver, and kelp/sea mustard intake as continuous variables in each tertile. Statistical significance was set at *p*-value < 0.05. The IBM SPSS Statistics 26 package (IBM, Armonk, NY, USA) was used for statistical analyses.

## 3. Results

### 3.1. General Characteristics According to Total Seaweed Consumption

The general characteristics of the study population according to the tertiles of total seaweed consumption are presented in Table 1. Compared with participants in the lowest tertile of total seaweed consumption, those in the highest tertile were younger with higher education levels, higher incomes and higher BMI, and they resided in urban areas (all *p*-values < 0.05). Those in the highest total seaweed consumption tertile were less likely to be current smokers, currently consuming alcohol, or physically active (all *p*-values < 0.05).

### 3.2. Dietary Intake and Clinical Characteristics According to Total Seaweed Consumption

The dietary intake and characteristics of the metabolic syndrome parameters according to tertiles of total seaweed consumption are shown in Table 2. Total seaweed consumption was significantly associated with dietary intake, with a higher intake of total energy, protein, fat, carbohydrates, and fiber in the highest tertile group (all *p*-values < 0.001). In addition, individuals in the highest tertile of total seaweed consumption had approximately seven times higher intakes of total seaweed, laver, and kelp/sea mustard than those in the lowest tertile (all *p*-values < 0.0001). In terms of the components of metabolic syndrome, there was a significant difference only in blood pressure according to the total seaweed consumption. Those in the highest total seaweed consumption tertile exhibited lower systolic (116.7 ± 16.4 vs. 118.4 ± 16.4 mmHg) and diastolic blood pressure (78.0 ± 11.0 vs. 78.8 ± 10.7 mmHg) (all *p*-values < 0.0001) than those in the lowest tertile group.

### 3.3. Association between Seaweed Consumption and the Odds of Developing Metabolic Syndrome and Its Components

The associations between total seaweed, laver, and kelp/sea mustard consumption and the prevalence of metabolic syndrome was analyzed (Table 3). Women in the highest tertile exhibited lower odds of developing metabolic syndrome than those in the lowest tertile of laver consumption (AOR = 0.70, 95% confidence interval (CI): 0.54–0.92; *p* < 0.05). However, total consumption of seaweed and kelp/sea mustard was not significantly associated with metabolic syndrome.

We further examined the odds of individual metabolic syndrome components according to the total consumption of seaweed, laver, and kelp/sea mustard (Table 4). Laver consumption was significantly associated with abdominal obesity in both men and women. Compared with the individuals in the lowest tertile of laver consumption, those in the highest tertile had reduced odds of abdominal obesity (AOR = 0.64, 95% CI: 0.42–0.98 for men; AOR = 0.53, 95% CI: 0.39–0.72 for women). However, no significant association was observed between total seaweed and kelp/sea mustard consumption and the individual components of metabolic syndrome.

## 4. Discussion

In the present study, women with higher laver consumption had lower odds of metabolic syndrome. Furthermore, high laver intake was associated with lower odds of abdominal obesity in both men and women. Consistent with our findings, waist circumference in Koreans aged 19–50 years was significantly reduced after 12 weeks of consumption of *Gelidium elegans* (red algae) [35]. Our findings also indicate that there is no association between the total seaweed intake and metabolic syndrome. However, inconsistent findings have been reported regarding the association between seaweed intake and metabolic syndrome. The consumption of 6 g/day of seaweed may be associated with improved parameters of metabolic syndrome, decreasing waist circumference and blood pressure in a non-seaweed-consuming Andean population [36]. Furthermore, iodine and seaweed intake results in low blood glucose, blood pressure, and triglyceride levels in post-menopausal Korean women [25]. Meanwhile, Korean men aged ≥30 years with increased seaweed intake, such as laver, had an increased risk of metabolic syndrome [37]. These inconsistent findings regarding seaweed intake and metabolic syndrome may be partially due to age differences in the target study populations. Furthermore, the rates of current smokers and those consuming alcohol were approximately double than those of our study participants. These differences may have influenced the contrasting findings.

According to a Japanese study, frequent consumption of seaweed is likely to reduce serum triglyceride levels in participants with abnormally high levels [38]. This may be partially due to the fact that seaweed-soluble dietary fibers are highly viscous and, thus, hinder the absorption of triglycerides [39]. In another study, rats that consumed seaweed after being stressed showed higher triglyceride levels than control rats [40]. These findings suggest that seaweed consumption does not improve serum triglyceride levels. In line with this, we found that seaweed consumption was not significantly associated with hypertriglyceridemia. In addition, we did not find any significant association between seaweed consumption and hyperglycemia. Contrary to our findings, previous studies have shown that low-molecular-weight polysaccharides obtained from laver via hydrogen peroxide decomposition have excellent hypoglycemic and α-amylase-inhibitory activities in rats [41]. Koreans aged 40–69 years who ate seaweed—especially kelp and sea mustard—had a reduced risk of diabetes [42]. Seaweed consumption reduces the risk of hyperglycemia, because protein hydrolysates and peptides derived from seaweed may lower blood-glucose levels [28].

Seaweed polysaccharides have antihypertensive effects in rats [43]. In a study on young children in Japan, the consumption of laver showed a diastolic-blood-pressure-lowering effect in boys [44]. Although several studies have reported significant relationship between blood pressure and seaweed consumption, no relationship was found between hypertension and seaweed consumption in the present study. The difference in the results of this study reflects the variations between the environments of Japan and Korea, which differ in terms of climate, sea environment, and lifestyle, including the consumption of specific foods. In addition, seaweed peptides, which are effective in preventing hyperglycemia, have been reported to exert antihypertensive effects [28].

In the present study, seaweed consumption was not significantly associated with low HDL cholesterol levels. In line with our findings, several studies have reported no association between seaweed consumption and HDL cholesterol levels [45,46]. In rats fed kelp-supplemented cabbage kimchi, the concentration of triglycerides decreased significantly. However, no significant changes were observed in HDL cholesterol levels [45]. A previous study showed that *Ecklonia cava*—a brown seaweed—improved LDL/HDL cholesterol ratios in diabetic rodents [47]. However, another study found that HDL cholesterol levels increased when rodents consumed a seaweed porphyran drink after consuming a high-cholesterol diet [48]. This difference in the results may be due to an inconsistent relationship between seaweed consumption and HDL-C, and these investigations are usually conducted in vivo, often in rats.

This study has several strengths. First, this is the first population-based study to investigate the association between seaweed, laver, kelp/sea mustard, and metabolic syndrome and its components. Second, we evaluated the independent association between seaweed consumption and metabolic syndrome by correcting for variables that could affect the results, such as age, alcohol consumption, smoking status, physical activity, and dietary intake of other foods.

The study also had some limitations. The mechanism by which seaweed consumption affects metabolic syndrome and its components has not yet been assessed. Furthermore, the composition of seaweed varies greatly depending on the harvesting region and growing conditions [49]. Because the growth conditions of seaweed differ from region to region, the present study did not demonstrate significant associations between seaweed consumption and metabolic syndrome and its components, except for abdominal obesity. Additionally, the causal relationship between seaweed consumption and metabolic syndrome could not be established, owing to the cross-sectional design of the present study.

## 5. Conclusions

Laver consumption was associated with lower odds of metabolic syndrome and abdominal obesity among middle-aged and elderly Korean adults. However, no association was found between total seaweed intake including laver and kelp/sea mustard—and metabolic syndrome. Our findings suggest that laver consumption is inversely associated with metabolic syndrome and abdominal obesity. Further studies are warranted to investigate the causal relationship between total seaweed, laver, and kelp/sea mustard intake and the risk of developing metabolic syndrome.

## Figures and Tables

**Table 1:** General characteristics according to tertiles of total seaweed consumption in the Korean Genome and Epidemiology Study (Ansan and Ansung).

Variables	Tertile of Total Seaweed Consumption			*p*-Value
	T1 (Lowest) (*n* = 1945)	T2 (*n* = 1628)	T3 (Highest) (*n* = 2204)	
Age (years)	51.3 ± 8.7 ^(1)^	49.9 ± 8.3	49.4 ± 8.1	<0.0001
Education				<0.0001
Elementary/middle school	1124 (57.8%)	750 (46.1%)	965 (43.8%)	
High school/technical college	657 (33.8%)	690 (42.4%)	939 (42.6%)	
University	164 (8.4%)	188 (11.5%)	300 (13.6%)	
Income (million KRW/month)				<0.0001
< 1	706 (36.3%)	392 (24.1%)	483 (21.9%)	
1–3	933 (48.0%)	845 (51.9%)	1143 (51.9%)	
> 3	306 (15.7%)	391 (24.0%)	578 (26.2%)	
Smoking status				0.02
None	1075 (55.3%)	962 (59.1%)	1323 (60.0%)	
Past	307 (15.8%)	247 (15.2%)	333 (15.1%)	
Current	563 (28.9%)	419 (25.7%)	548 (24.9%)	
Alcohol consumption				0.015
None	810 (41.6%)	718 (44.1%)	1027 (46.6%)	
Past	126 (6.5%)	90 (5.5%)	110 (5.0%)	
Current	1009 (51.9%)	820 (50.4%)	1067 (48.4%)	
Region				<0.0001
Ansung (rural)	852 (43.8%)	441 (27.1%)	558 (25.3%)	
Ansan (urban)	1093 (56.2%)	1187 (72.9%)	1646 (74.7%)	
Physical activity (MET-h/d)	23.6 ± 14.9	21.3 ± 13.1	21.7 ± 13.1	<0.0001
Body mass index (kg/m^2^)	24.1 ± 3.0	24.3 ± 3.0	24.6 ± 3.0	<0.0001

MET: metabolic equivalent of task. ^(1)^ Values are presented as the mean ± standard deviation.

**Table 2:** Dietary intake and clinical characteristics according to tertiles of total seaweed consumption in the Korean Genome and Epidemiology Study (Ansan and Ansung).

Variables	Tertile of Total Seaweed Consumption			*p*-Value
	Tertile 1 (Lowest)	Tertile 2	Tertile 3 (Highest)	
Dietary intake				
Total energy (kcal)	1748.6 ± 575.3 ^(1)^	1912 ± 525.1	2182.9 ± 758.3	<0.0001
Protein (g)	55.5 ± 22.3	65.2 ± 21.2	80.3 ± 35.1	<0.0001
Fat (g)	26.8 ± 16.9	32.7 ± 16.0	41.2 ± 24.9	<0.0001
Carbohydrates (g)	315.5 ± 98.6	334.3 ± 92.0	368.9 ± 121.8	<0.0001
Dietary fiber (g)	5.9 ± 2.8	6.7 ± 2.7	8.1 ± 3.8	<0.0001
Total seaweed (g)	0.5 ± 0.3	1.4 ± 0.3	3.6 ± 2.2	<0.0001
Laver (g)	0.3 ± 0.2	0.8 ± 0.4	2.0 ± 1.4	<0.0001
Kelp/sea mustard (g)	0.2 ± 0.2	0.7 ± 0.4	1.6 ± 1.7	<0.0001
Components of metabolic syndrome				
Waist circumference (cm)	81.4 ± 8.5	81.0 ± 8.5	81.2 ± 8.6	0.157
Fasting glucose (mg/dL)	86.4 ± 21.8	86.6 ± 19.3	87.7 ± 22.9	0.095
Triglycerides (mg/dL)	157.7 ± 104.2	156.8 ± 98.1	151.6 ± 101.1	0.117
HDL cholesterol (mg/dL)	45.1 ± 10.0	45.0 ± 9.9	45.3 ± 10.1	0.634
Systolic blood pressure (mmHg)	118.4 ± 16.4	116.2 ± 16.0	116.7 ± 16.4	<0.0001
Diastolic blood pressure (mmHg)	78.8 ± 10.7	77.4 ± 10.8	78.0 ± 11.0	<0.0001

HDL cholesterol: high-density lipoprotein cholesterol. ^(1)^ Values are presented as the mean ± standard deviation.

**Table 3:** Odds ratios (ORs) and 95% confidence intervals (CIs) of metabolic syndrome according to tertiles of total seaweed, laver, and kelp/sea mustard consumption in the Korean Genome and Epidemiology Study (Ansan and Ansung).

	Men				Women			
	Tertile of Seaweed Consumption			*p*-Value for Trend ^(2)^	Tertile of Seaweed Consumption			*p*-Value for Trend
	T1 (Lowest)	T2	T3 (Highest)		T1 (Lowest)	T2	T3 (Highest)	
	OR (95% CI) ^(1)^	OR (95% CI)	OR (95% CI)		OR (95% CI)	OR (95% CI)	OR (95% CI)	
Total seaweed								
Cases/Total (*n*)	218/1012	192/861	224/963		326/988	264/958	275/995	
Crude model	1.00	1.05 (0.84–1.31)	1.10 (0.89–1.36)	0.36	1.00	0.77 (0.64–0.94)	0.78 (0.64–0.94)	0.025
Adjusted model ^(1)^	1.00	0.98 (0.76–1.26)	0.84 (0.64–1.10)	0.188	1.00	0.90 (0.71–1.13)	0.85 (0.67–1.09)	0.244
Laver								
Cases/Total (*n*)	142/625	299/1342	193/869		246/690	371/1323	248/928	
Crude model	1.00	0.98 (0.78–1.22)	0.97 (0.76–1.24)	0.860	1.00	0.70 (0.58–0.86)	0.66 (0.53–0.82)	0.003
Adjusted model	1.00	1.09 (0.83–1.42)	0.78 (0.57–1.06)	0.016	1.00	0.84 (0.66–1.06)	0.70 (0.54–0.92)	0.014
Kelp/sea mustard								
Cases/Total (*n*)	231/1095	150/673	253/1068		261/813	221/741	383/1387	
Crude model	1.00	1.07 (0.85–1.35)	1.16 (0.95–1.42)	0.150	1.00	0.90 (0.73–1.12)	0.81 (0.67–0.97)	0.028
Adjusted model	1.00	1.08 (0.83–1.41)	1.11 (0.87–1.41)	0.460	1.00	0.99 (0.77–1.27)	0.92 (0.73–1.16)	0.446

^(1)^ Adjusted for age, region, alcohol consumption, smoking status, education level, income level, BMI, physical activity level, total energy intake, and protein, fat, carbohydrate, and fiber intake; ^(2)^ *p* for trend was calculated by using the median value of the seaweed intake in each category.

**Table 4:** Odds ratios (ORs) and 95% confidence intervals (CIs) of individual metabolic syndrome components according to tertiles of total seaweed, laver, and kelp/sea mustard consumption in the Korean Genome and Epidemiology Study (Ansan and Ansung).

	Men				Women			
	Tertile of Seaweed Consumption			*p*-Value for Trend ^(2)^	Tertile of Seaweed Consumption			*p*-Value for Trend
	T1 (Lowest)	T2	T3 (Highest)		T1 (Lowest)	T2	T3 (Highest)	
	OR (95% CI) ^(1)^	OR (95% CI)	OR (95% CI)		OR (95% CI)	OR (95% CI)	OR (95% CI)	
Total seaweed								
Abdominal obesity	1.00	0.99 (0.69–1.41)	0.83 (0.58–1.21)	0.301	1.00	0.85 (0.66–1.11)	0.91 (0.68–1.20)	0.641
Elevated triglycerides	1.00	1.10 (0.90–1.34)	0.89 (0.72–1.10)	0.207	1.00	0.93 (0.75–1.24)	0.90 (0.72–1.13)	0.421
Elevated fasting glucose	1.00	0.82 (0.51–1.31)	0.94 (0.59–1.49)	0.915	1.00	0.86 (0.47–1.57)	0.86 (0.46–1.61)	0.709
Elevated blood pressure	1.00	0.85 (0.70–1.03)	0.98 (0.80–1.21)	0.967	1.00	0.86 (0.70–1.05)	0.93 (0.75–1.15)	0.696
Low HDL cholesterol	1.00	0.92 (0.75–1.13)	0.83 (0.67–1.03)	0.096	1.00	0.96 (0.78–1.17)	0.94 (0.76–1.17)	0.610
Laver								
Abdominal obesity	1.00	0.79 (0.54–1.15)	0.64 (0.42–0.98)	0.058	1.00	0.62 (0.47–0.81)	0.53 (0.39–0.72)	0.001
Elevated triglycerides	1.00	1.09 (0.88–1.35)	0.88 (0.69–1.12)	0.065	1.00	0.93 (0.75–1.15)	0.81 (0.64–1.04)	0.089
Elevated fasting glucose	1.00	0.73 (0.45–1.19)	0.79 (0.47–1.34)	0.748	1.00	0.99 (0.51–1.94)	1.26 (0.63–2.52)	0.385
Elevated blood pressure	1.00	0.91 (0.74–1.12)	0.97(0.76–1.22)	0.884	1.00	1.04 (0.84–1.28)	1.09 (0.86–1.38)	0.496
Low HDL cholesterol	1.00	1.10 (0.88–1.37)	0.81 (0.63–1.04)	0.008	1.00	0.89 (0.72–1.10)	0.92 (0.73–1.17)	0.780
Kelp/sea mustard								
Abdominal obesity	1.00	1.27 (0.87–1.81)	1.08 (0.77–1.52)	0.864	1.00	0.76 (0.56–1.00)	1.05 (0.81–1.37)	0.236
Elevated triglycerides	1.00	0.91 (0.74–1.12)	0.96 (0.79–1.16)	0.817	1.00	1.05 (0.83–1.32)	0.94 (0.76–1.16)	0.383
Elevated fasting glucose	1.00	1.17 (0.73–1.89)	1.14 (0.74–1.75)	0.660	1.00	1.70 (0.90–3.21)	0.79 (0.41–1.51)	0.103
Elevated blood pressure	1.00	0.80 (0.65–0.97)	0.89 (0.74–1.08)	0.437	1.00	1.01 (0.81–1.27)	0.88 (0.72–1.08)	0.155
Low HDL cholesterol	1.00	1.11 (0.90–1.37)	1.04 (0.86–1.27)	0.824	1.00	1.04 (0.83–1.29)	1.01 (0.83–1.23)	0.992

HDL cholesterol: high-density lipoprotein cholesterol. ^(1)^ Adjusted for age, region, alcohol consumption, smoking status, education level, income level, physical activity level, total energy intake, and protein, fat, carbohydrate, and fiber intake; ^(2)^ *p* for trend was calculated using the median value of seaweed intake in each category.

## Data Availability

The data underlying the results of our study are not publicly available because of the data policy of KoGES. Data are available from the Division of Genetic Epidemiology and Health Index, NIH, Korea Centers for Disease Control and Prevention, for researchers who meet the criteria for access to confidential data.
